# Involvement of the FTO A > T polymorphism in body composition and lipid profile changes after aerobic training in adults with overweight and obesity

**DOI:** 10.1111/dom.70578

**Published:** 2026-02-18

**Authors:** Herik Fonseca, Glebia Cardoso, George Araújo, Eriklys Barreto, Mateus Ribeiro, Ana Pereira, Cecília Marinho, Bruno Sousa, Victor Ferreira, Lydiane Toscano, Raquel Silva, Thais Assis, Darlene Persuhn, Alexandre Silva

**Affiliations:** ^1^ Postgraduate Program in Physical Education, Center for Health Sciences Federal University of Paraíba João Pessoa Brazil; ^2^ Laboratory of Applied Studies in Physical Training for Performance and Health, Department of Physical Education Federal University of Paraíba João Pessoa Brazil; ^3^ Postgraduate Program in Health and Society State University of Rio Grande do Norte Mossoró Brazil; ^4^ Postgraduate Program in Movement Sciences Federal University of Piauí Teresina Brazil; ^5^ Postgraduate Program in Nutritional Sciences, Center for Health Sciences Federal University of Paraíba João Pessoa Brazil; ^6^ Department of Physiotherapy Federal University of Paraíba João Pessoa Brazil

**Keywords:** body composition, body mass loss, physical training, polymorphism, weight loss

## 1 BACKGROUND

Aerobic exercise training is a recommended non‐pharmacological strategy for obesity management.^1^, ^2^ However, weight loss achieved through exercise training is considered clinically modest, typically ranging from 2 to 3 kg after at least 3 months.^3^ Genetic polymorphisms have been proposed to explain this phenomenon and several are associated with obesity, energy metabolism, lipid biology, and adipogenesis.^4^ However, evidence for exercise‐induced weight loss has been demonstrated only for peroxisome proliferator‐activated receptor alpha (PPAR‐α)^5^ and the coactivator (PPARGC1A).^6^

Individuals homozygous for the A allele of the fat mass and obesity–associated gene (FTO; rs9939609) exhibit approximately 3 kg higher body weight and a 67% greater risk of obesity compared with non‐carriers or heterozygotes.^7^ A meta‐analysis has demonstrated strong associations between FTO polymorphisms, including rs9939609, and obesity.^8^ Based on this evidence, we hypothesized that FTO rs9939609 influences responsiveness to exercise‐induced weight loss. Therefore, this study investigated the effect of the FTO rs9939609 polymorphism on weight loss and lipid profile changes induced by a 12‐week aerobic training program in individuals with overweight and obesity.

## 2 METHODS

Adults with overweight or obesity were randomly allocated in a 3:1 ratio to an experimental group (EXG) that completed a 12‐week moderate‐intensity aerobic training program (walking/running with progressive frequency, duration, and intensity), or to a control group (CG), which remained sedentary throughout the study. Body composition (dual‐energy X‐ray absorptiometry [DXA]), dietary intake, and cardiopulmonary exercise testing were assessed at baseline and after the intervention (see Data S1, Supporting Information). Buccal mucosa samples were collected for genotyping of the FTO rs9939609 polymorphism. EXG was subsequently subdivided according to genotypes for comparison of training outcomes.

## 3 STATISTICAL ANALYSIS

Data are mean ± standard deviation. Normality was assessed using the Kolmogorov–Smirnov test. One‐way ANOVA was used to compare baseline characteristics, and two‐way repeated‐measures ANOVA was used to assess training effects on body composition. Analyses were performed using JAMOVI software (version 2.3), with statistical significance set at *p* < 0.05.

## 4 RESULTS

Eighty‐eight participants completed the intervention. In the exercise group, 70 individuals were successfully genotyped, resulting in 21 with the TT genotype and 49 with the TA genotype (AA genotype was not observed). Eighteen participants comprised the control group, and the CG was younger than the EXG (Table S1). Total energy and macronutrient intake were similar across genotypes and the control group at baseline, with no significant changes during the intervention, except for an increase in carbohydrate intake at week 12 among TA carriers (Table S2).

Training effects are presented in Table 1. While CG demonstrated no changes, EXG showed a significant increase in VO_2max_ and reductions in fat mass, body fat percentage, waist circumference, and hip circumference (all *p* < 0.05) after the intervention. EXG exhibited significant reductions in total cholesterol and LDL cholesterol (both *p* < 0.05).

**TABLE 1:** Effects of a 12‐week aerobic training program on cardiorespiratory fitness, lipid profile, and body composition.

	Exercise group (*n* = 70)			Control group (*n* = 18)			TT (*n* = 21)			TA (*n* = 49)		
	Pre	Post	Δ	Pre	Post	Δ	Pre	Post	Δ	Pre	Post	Δ
VO_2_max	27.9 ± 7.0	33.5 ± 9.1^a^, ^b^	5.5 ± 5.4^b^	24.7 ± 4.9	25.7 ± 3.7	0.2 ± 2.9	28.6 ± 6.7	34.3 ± 9.0^a^	5.6 ± 6.1	27.7 ± 7.1	33.2 ± 9.3^a^	5.5 ± 5.1
Col (mg/dL)	200.0 ± 47.2	185.0 ± 42.0^a^	−14.9 ± 35.8^b^	186.0 ± 38.0	196.0 ± 52.6	9.9 ± 41.2	193.0 ± 34.1	175.0 ± 43.0	−17.0 ± 27.3	203.0 ± 51.8	190.0 ± 41.3^a^	−13.7 ± 39.0
LDL (mg/dL)	131.0 ± 39.1	119.0 ± 37.9^a^	−12.5 ± 34.2	125.0 ± 33.4	130.0 ± 44.6	5.5 ± 42.6	126.0 ± 31.0	108.0 ± 40.6	−18.0 ± 30.6	134.0 ± 42.2	123.0 ± 36.2	−10.7 ± 34.3
HDL (mg/dL)	40.0 ± 10.9	41.6 ± 13.1	2.3 ± 8.3	34.3 ± 11.2	36.7 ± 7.8	1.0 ± 9.0	40.7 ± 10.7	41.8 ± 12.5	1.1 ± 7.7	41.1 ± 10.1	41.6 ± 13.4	1.5 ± 8.6
TRG (mg/dL)	142.0 ± 90.5	126.0 ± 71.6	−16.7 ± 56.8	135.0 ± 72.7	147.0 ± 82.1	12.2 ± 58.2	130.0 ± 59.5	127.0 ± 57.9	−2.6 ± 45.8	147.0 ± 101.0	125.0 ± 77.2^a^	−22.0 ± 60.3
BW (kg)	84.5 ± 11.6	83.7 ± 11.6	−0.7 ± 2.4	83.6 ± 12.5	84.0 ± 12.5	0.2 ± 2.5	80.2 ± 8.8	80.1 ± 8.9	−0.4 ± 1.9	86.2 ± 7.6	85.3 ± 12.4	−0.9 ± 2.6
BMI (kg/m^2^)	31.3 ± 3.3	31.1 ± 3.4	−0.2 ± 1.0	31.1 ± 3.3	31.1 ± 3.3	0.0 ± 0.9	30.4 ± 3.1	30.3 ± 3.5	−0.1 ± 0.7	31.7 ± 3.3	31.4 ± 3.3	−0.3 ± 1.0
FM (kg)	37.3 ± 6.8	36.2 ± 6.8^a^	−1.1 ± 1.9	39.0 ± 7.5	38.8 ± 8.2	−0.2 ± 1.3	36.1 ± 5.9	35.0 ± 6.4	−1.1 ± 1.9	37.9 ± 6.6	36.7 ± 7.0^a^	−1.1 ± 2.0
% BF	44.9 ± 6.3	43.8 ± 6.4^a^	−1.1 ± 1.6	45.5 ± 6.9	45.1 ± 6.8	−0.3 ± 1.4	45.5 ± 5.2	44.3 ± 5.6^a^	−1.1 ± 1.7	44.7 ± 6.8	43.5 ± 6.7^a^	−1.1 ± 1.5
FFM (kg)	44.3 ± 9.5	44.8 ± 9.3	−0.7 ± 1.7	42.5 ± 7.6	43.0 ± 7.0	2.4 ± 1.9	41.5 ± 6.9	42.1 ± 7.1	0.6 ± 1.2	45.5 ± 10.3	45.9 ± 9.9	0.4 ± 1.8
WC (cm)	94.9 ± 8.7	92.9 ± 8.8^a^	1.9 ± 3.1^b^	95.5 ± 8.9	94.7 ± 9.7	0.0 ± 3.5	95.2 ± 9.3	92.3 ± 9.8^a^	−2.8 ± 2.3	94.8 ± 8.6	93.2 ± 8.4^a^	−1.5 ± 3.3
HC (cm)	111.0 ± 7.0	109.0 ± 6.6^a^	−2.5 ± 4.0^b^	111.0 ± 6.8	111.0 ± 7.4	−0.2 ± 4.5	111.0 ± 5.6	109.0 ± 6.4	−1.4 ± 3.1	111.0 ± 7.6	108.0 ± 6.7^a^	−3.0 ± 4.4

*Note*: Data are presented as mean ± standard deviation for the exercise and control groups and according to FTO rs9939609 genotype (TT and TA).

Abbreviations: %BF, body fat percentage; BMI, body mass index; BW, body weight; FFM, fat‐free mass; FM, fat mass; HC, hip circumference; HDL, high‐density lipoprotein cholesterol; LDL, low‐density lipoprotein cholesterol; TC, total cholesterol; TG, triglycerides; WC, waist circumference.

^a^ Within‐group differences between pre‐ and post‐intervention.

^b^ Between‐group differences at post‐intervention (repeated‐measures ANOVA, *p* < 0.05).

Genotype‐stratified analyses (Table 1) indicated that reductions in fat mass and hip circumference (both *p* < 0.001) occurred only among TA carriers, whereas both TT and TA groups showed significant and similar reductions in body fat percentage and waist circumference (both *p* < 0.001). Reductions in total cholesterol and triglycerides were significant only among TA carriers (both *p* < 0.001).

Sex‐specific analyses were restricted to women (Table 2) due to insufficient sample size in men. The exercise group showed significant reductions over time in fat mass, body fat percentage, waist and hip circumference, as well as an increase in lean mass (all *p* < 0.05). No significant changes were observed in the control group. Although these changes occurred as time effects, only waist circumference differed between groups after the intervention (time × group interaction). In genotype‐stratified analyses among women, the TA group showed significant reductions in fat mass, body fat percentage, and hip circumference (all *p* < 0.001), whereas the TT group showed a significant reduction only in waist circumference (*p* < 0.001). Both genotypes exhibited significant reductions in body fat percentage over time (*p* < 0.001), with no significant time × group interactions and no significant changes in lipid profile.

**TABLE 2:** Effects of a 12‐week aerobic training program on VO_2max_, lipid profile, and body composition in women.

	Exercise group (*n* = 53)			Control group (*n* = 15)			TT (18)			TA (35)		
	Pre	Post	Δ	Pre	Post	Δ	Pre	Post	Δ	Pre	Post	Δ
VO_2_max	26.2 ± 6.4	30.7 ± 3.6^a^	4.6 ± 3.6^a^	24.7 ± 4.7	25.6 ± 3.8	0.6 ± 2.8	25.6 ± 5.8	34.3 ± 6.5^a^	4.6 ± 4.0	27.4 ± 6.6	33.2 ± 7.3^a^	4.7 ± 3.5
Col (mg/dL)	198.0 ± 41.5	185.0 ± 41.8	−12.9 ± 36.5	190.0 ± 40.1	200.0 ± 50.8	9.9 ± 45.1	200.0 ± 31.1	182.0 ± 43.2	−18.3 ± 27.0	197.0 ± 46.3	187.0 ± 41.5	−10.1 ± 40.6
LDL (mg/dL)	130.0 ± 38.1	118.0 ± 37.9	−12.3 ± 21.4	126.0 ± 36.1	132.0 ± 48.9	6.1 ± 42.6	131.0 ± 31.0	112.0 ± 42.6	−19.4 ± 31.0	130.0 ± 42.2	121.0 ± 35.8	−8.7 ± 36.2
HDL (mg/dL)	42.3 ± 11.3	43.6 ± 13.5	1.3 ± 8.8	34.1 ± 12.3	37.6 ± 8.2	2.5 ± 9.6	41.4 ± 10.9	43.8 ± 12.3	2.4 ± 7.0	42.7 ± 11.6	43.5 ± 14.3	0.7 ± 9.6
TRG (mg/dL)	127.0 ± 68.2	117.0 ± 63.2	−9.7 ± 49.8	144.0 ± 75.7	151.0 ± 89.4	6.4 ± 62.2	137.0 ± 61.0	130.0 ± 60.6	−6.8 ± 45.8	121.0 ± 71.9	110.0 ± 64.6	−11.2 ± 52.3
BW (kg)	81.0 ± 10.0	80.5 ± 9.6	−0.5 ± 2.3	81.2 ± 9.3	81.6 ± 9.6	0.4 ± 2.6	79.1 ± 7.9	78.9 ± 8.3	−0.2 ± 1.7	82.0 ± 10.3	85.3 ± 10.3	−0.6 ± 2.5
BMI (kg/m^2^)	31.2 ± 3.3	31.0 ± 3.4	−0.1 ± 0.9	30.9 ± 3.10	31.1 ± 3.0	0.0 ± 1.0	30.5 ± 3.4	30.5 ± 3.7	0.3 ± 0.7	31.3 ± 3.3	31.2 ± 3.3	−0.3 ± 0.9
FM (kg)	38.0 ± 6.6	37.0 ± 6.3^a^	−1.0 ± 1.7	38.8 ± 5.0	38.6 ± 6.0	−0.1 ± 1.4	36.4 ± 6.3	35.5 ± 6.7	−0.9 ± 1.7	38.9 ± 6.7	37.8 ± 6.8^a^	−1.1 ± 1.8
% FM	47.6 ± 4.4	46.4 ± 4.4^a^	−1.1 ± 1.4	46.5 ± 6.7	46.1 ± 6.1	−0.3 ± 1.5	46.8 ± 6.3	45.8 ± 4.6^a^	−0.8 ± 1.7	48.0 ± 6.7	46.7 ± 4.4^a^	−1.2 ± 1.3
FFM (kg)	39.9 ± 5.0	40.5 ± 4.9^a^	0.6 ± 4.9	40.5 ± 6.7	41.0 ± 5.8	0.4 ± 5.8	39.4 ± 4.5	40.0 ± 4.8	0.5 ± 1.3	40.1 ± 5.2	40.8 ± 9.9	0.6 ± 1.7
WC (cm)	111.0 ± 7.4	91.6 ± 8.1^a^	1.7 ± 3.0^b^	92.9 ± 7.2	93.2 ± 8.5	0.6 ± 3.8	95.8 ± 8.8	93.0 ± 9.4^a^	−2.8 ± 2.4	92.0 ± 7.4	90.8 ± 7.5	−1.1 ± 3.1
HC (cm)	111.0 ± 7.0	109.0 ± 6.7^a^	−2.0 ± 4.4	111.0 ± 6.8	111.0 ± 6.8	0.0 ± 3.6	111.0 ± 5.6	110.0 ± 6.4	−1.3 ± 6.4	112.0 ± 8.2	109.0 ± 7.0^a^	−3.0 ± 7.0

*Note*: Data are presented as mean ± standard deviation for women in the exercise and control groups and according to FTO rs9939609 genotype (TT and TA).

Abbreviations: %BF, percentage of body fat; BMI, body mass index; BW, body weight; FFM, fat‐free mass; FM, fat mass; HC, hip circumference; HDL, high‐density lipoprotein cholesterol; LDL, low‐density lipoprotein cholesterol; TC, total cholesterol; TG, triglycerides; WC, waist circumference.

^a^ Within‐group differences between pre‐ and post‐intervention.

^b^ Between‐group differences at post‐intervention (repeated‐measures ANOVA, *p* < 0.05).

## 5 DISCUSSION

Aerobic training improved cardiorespiratory fitness and promoted statistically significant but clinically modest reductions in fat mass, given that the reduction was 1.1%, below the ≥5% threshold considered clinically meaningful.^1^ These findings align with previous evidence highlighting the modest magnitude of exercise‐induced weight loss.^9^

The limited magnitude of fat loss following aerobic training has been attributed to compensatory mechanisms, including reductions in energy expenditure and increases in energy intake.^10^ However, these mechanisms do not fully explain the limited weight loss, suggesting additional factors.

Our laboratory has investigated genetic influences as one such factor. Previous studies demonstrated that polymorphisms in the PPARGC1A are associated with variability in fat loss responses to exercise training.^6^ Our data are consistent with findings from Mazur et al.,^6^ who reported an influence of this same polymorphism in a training program similar to ours. However, considering that the human genome comprises approximately 25 000 genes, PPARGC1A‐related effects likely represent only an initial step, and other genetic polymorphisms remain to be investigated.

To support the search for additional variants, we focused on polymorphisms implicated in energy metabolism, body composition, and appetite regulation.^11^, ^12^ Among these, the fat mass and obesity‐associated gene (FTO), as suggested by its name, has been most consistently linked to obesity. In the only study that investigated the FTO rs9939609 in response to aerobic training, no significant changes in body composition were observed; however, that study included young and normal‐weight participants.^12^

In the present study, reductions in hip circumference, absolute fat mass, total cholesterol, and triglycerides were observed only among TA carriers over time. Among women, this genotype‐specific responsiveness was maintained for hip circumference and absolute fat mass, whereas lipid changes occurred only as time effects. These findings support further investigation of the influence of the FTO rs9939609 on metabolic and body composition responses to exercise training. Nevertheless, the results should be interpreted in light of the statistical and methodological aspects of the present study. One important limitation is the absence of the AA genotype, which may reflect population characteristics or insufficient sample size. Notably, the AA genotype is associated with a higher risk of obesity. Despite this limitation, the presence of the A allele modestly facilitated exercise‐induced weight loss, consistent with observational evidence suggesting greater benefits from lifestyle interventions among A‐allele carriers.^10^, ^11^ Although groups were statistically similar at baseline, descriptive values for variables responsive exclusively in the TA group were higher before the intervention. This observation should be considered in future studies, as it may indicate normalization of initially higher values.

The data from the present study provide a basis for further investigation of the influence of FTO rs9939609 on exercise‐induced weight loss. Such investigations should include larger sample sizes to allow the inclusion of individuals with the AA genotype or involve collaboration with laboratories from other ethnic groups in which the frequency of the AA polymorphism is higher.

## 6 CONCLUSION

Carriers of the FTO rs9939609 TA genotype exhibited greater changes across multiple body composition and lipid profile variables compared with TT carriers after aerobic training, with changes observed only as time effects and no significant genotype × time interactions. The absence of AA homozygotes in the study population may have limited the magnitude of the observed responses. These findings suggest the need for larger, multicentre studies, as AA genotype prevalence appears very low in the population investigated.

## AUTHOR CONTRIBUTIONS

H.F. and A.S. conceived the manuscript, agreed on the content, contributed to the drafting and editing of the manuscript, and approved the final version. G.C., E.B., M.R., A.P., T.A., B.S., V.F., J.M., and D.P. contributed to the drafting and editing of the manuscript and approved the final version.

## CONFLICT OF INTEREST STATEMENT

The authors declare no conflicts of interest.

## Supporting information


**Data S1.** Supporting Information.

## Data Availability

The data that support the findings of this study are available from the corresponding author upon reasonable request.
