# Supplementary material for: Involvement of the FTO A > T polymorphism in body composition and lipid profile changes after aerobic training in adults with overweight and obesity
Source: Diabetes Obes Metab. 2026 Feb 18;28(5):4361–5. doi: 10.1111/dom.70578 (PMC13071253; doi:10.1111/dom.70578)
Supplement: Supplementary file 1 — Data S1. Supporting Information. [file DOM-28-4361-s001.docx]

**Methods**

Randomized controlled clinical study. The sample size was determined based on data from the study by Kim and Jung (2014), where a physical training program led to a fat loss of 2.18±1.96% (effect size of 0.66). Adopting a significance level (alpha) of 0.05 and a power of 80%, it was estimated that the minimum sample size required for the present study would be 16 people with obesity (GPower 3.1 software, Franz Faul, University of Kiel, Germany). However, considering that participants would be subdivided into genotypic groups, the study continued until at least 16 cases were found in the allele T group, which is less common in the population of the country where this study was conducted (18). Thus, the study was concluded with 21 participants in the TT group, 49 participants in the TA and AA groups combined, and 18 participants in the control group.

Inclusion criteria: Adults (20 to 45 years old) of both sexes, who had been sedentary for at least six months (<150 minutes of moderate to vigorous physical activity per week)^18^, presented with overweight or obesity (BMI between 25 kg/m² and 39.9 kg/m²) (WHO, 2022), had not experienced weight change (more than 5 kg) in the three months prior to study. Participants could not smoke or be alcoholics (maximum 2 drinks/day), nor could they consume supplements and/or medications that affect weight loss or gain. Additionally, they should not have a history of chronic, metabolic, or bone diseases that could influence participation, and women could not be menopausal or exhibit symptoms related to menopause. Moreover, participants needed to be classified as fit for aerobic physical training based on an ergometric test conducted before the intervention.

Participants who missed at least 25% of the total training program or two consecutive weeks, started medication treatment, supplementation, dietary protocol, and/or another physical training program during the intervention period, or presented any osteoarticular injury that hindered continuity, would be excluded from the study.

The study was conducted in accordance with the Helsinki declaration. The project was approved by the local Research Ethics Committee under protocol number 1.91.304 and CAAE: 65924917.5.0000.5188. After being informed about all procedures, risks, and benefits, volunteers were instructed to assign the Informed Consent Form.

**Aerobic capacity**

To determine the exercise intensity, aerobic capacity was assessed using ergospirometry (Metalyzer 3B - Cortex, Leipzig, Germany) on a treadmill (Centurion-200, Micromed, Brasília, Brazil), following the Bruce ramp protocol (8-12 minutes). Criteria for test termination adhered to the guidelines of Guazzi et al.^19^ , including maximal fatigue and maximum heart rate. The test would be halted if any participant experienced angina or dyspnea.

**Training protocol**

Before the training sessions, participants underwent a two-week adaptation period in the modality, which consisted of two training sessions per week, lasting 30 to 40 minutes each, at 50% of their maximum heart rate (HRmax). The aerobic training program (walking/running) starting at 40 minutes in the first week. From the second to the third week, the duration increased to 50 minutes, and from the fourth week until the end of the training period, sessions lasted 60 minutes. In the first week of formal training, the intensity was increased to 60% of HRmax, progressing to 75% in the final weeks of the training period. Intensity was monitored using a heart rate monitor (Polar®, model FT1, Polar Electro Oy, Kempele, Finland). The control group continued attending stretching classes once a week, each session lasting 60 minutes.

**Body composition**

For the assessment of body composition, Dual-Energy X-ray Absorptiometry (DXA) was employed. Measurement of criterion standards such as body fat percentage and lean body mass was obtained through a whole-body scan using the LUNAR ADVANCE DF+ 13.4038 Radiation device (GE LUNAR CORPORATION/USA). To assess body mass, a scale (Sanny®, São Bernardo do Campo - São Paulo, Brazil) was utilized. Circumferences were measured by a trained professional using a 0.1 cm steel anthropometric tape, recording waist, abdomen, and hip circumferences.

**Eating habits**

Nutritional assessments of dietary intake were conducted by an experienced nutritionist, involving three recalls per assessment period (two weekdays and one weekend day), using the 24-hour dietary recall (R24h) method recommended by the Dietary Reference Intakes (DRI)  ^20^ . For calculations of calorie intake and macro- and micronutrient consumption, the AVANUTRI software, version 4.0 (Avanutri & Nutrição Serviços de Informática, Três Rios-RJ, Brazil), was employed. Participants were instructed not to change their dietary habits.

**Collection and Analysis of Genetic Material**

Samples of oral cells were extracted via gargling for 60 seconds with 5ml of 3% sucrose solution. The resulting gargle content was transferred to a 15ml tube, followed by the addition of 3ml of a TNE solution (17mM Tris-HCl at pH 8.0, 50 mM NaCl, and 7mM EDTA), diluted to 66% with autoclaved distilled water and alcohol. The genotyping of the FTO gene polymorphism A>T was conducted using DNA amplification by PCR-RFLP (SAIKI et al., 1985). The polymorphism was amplified using the primers: (5'-AACTGGCTCTTGAATGAAATAGGATTCAGA-3') and (5'-AGAGTAACAGAGACTATCCAAGTGCAGTAC-3'). The PCR mixture was prepared with a 1x concentration of amplification mix (GoTaq® Green, Promega Master Mix), 2 pmol of each primer (Invitrogen, Carlsbad, CA, USA), 1 μl of the extracted sample, with a final volume of 15 μl completed with autoclaved Milli-Q water. In the thermocycler, the samples underwent cycles of denaturation, annealing, and extension at temperatures of 61°C and 72°C, respectively.

The PCR reaction was incubated at 94 °C for 5 min, followed by 20 cycles at 94 °C for 45 s, 61 °C for 45 s (decreasing by 0.5 °C per cycle), and 72 °C for 45 s. This was followed by 15 additional cycles at 94 °C for 45 s, 51 °C for 45 s, and 72 °C for 45 s, with a final extension at 72 °C for 10 min. The amplified products were then digested with 2 U of ScaI (New England Biolabs, Hitchin, UK) at 37 °C for 16 h. Digestion products were separated by electrophoresis on 15% polyacrylamide gels and visualized by silver nitrate staining. Fragment patterns indicated the presence of the A allele when 154- and 28-bp fragments were observed, whereas the T allele was identified by the undigested 182-bp fragment, as previously described (López-Bermejo et al., 2008).

**Statistical analysis**

Data are presented as mean ± standard deviation. Normality and homogeneity were assessed using the Kolmogorov-Smirnov and Levene test. Two-way ANOVA for repeated measures was employed to assess differences in the effect of training on body composition. This allowed for comparison of intergroup and intragroup results using the delta (Δ = post-pre). Repeated measures ANOVA with Greenhouse-Geisser correction was employed to analyze the pattern of caloric intake. Data analysis was performed using JAMOVI statistical software (version 2.3), with statistical significance set at p ≤ 0.05.

**Supplementary Figure 1.**

**Supplementary Table 1.** Baseline characteristics of participants according to intervention group and FTO rs9939609 genotype

|  | Exercise Group  (n=70) | Control Group  (n=18) | p | TT | TA |  |
| --- | --- | --- | --- | --- | --- | --- |
|  |  |  |  | (n=21) | (n=49) | p |
| Age (yrs) | 35.1 ± 8.91 | 29.1 ± 8.39* | 0.01 | 36.5 ± 7.90 | 34.4 ± 9.32 | 0.37 |
| AF (min/wk) | 59.7 ± 38.5 | 61.9 ± 33.80 | 0.83 | 60.2 ± 41.1 | 59.5 ± 37.5 | 0.94 |
| Training adherence | 84.7 ± 7.91 |  |  | 85.4 ± 8.80 | 84.4 ± 7.58 | 0.65 |
| IMC (kg/m^2^) | 31.3 ± 3.28 | 31.1 ± 3.31 | 0.99 | 30.4 ± 3.13 | 31.7 ± 3.29 | 0.68 |

Data are presented as mean ± standard deviation for the exercise and control groups and according to FTO rs9939609 genotype. PA: physical activity. * indicates between-group differences at baseline (p < 0.05).

**Supplementary Table 2.** Macronutrient and dietary intake during the 12-week intervention according to FTO rs9939609 genotype

| Nutrition | TT (n=21) | | | | | | | p | | TA (n=49) | | | | | | | p |  |
| --- | --- | --- | --- | --- | --- | --- | --- | --- | --- | --- | --- | --- | --- | --- | --- | --- | --- | --- |
|  | | Pre | | 6^th^ wk | | Post | |  | | Pre | | 6^th^ wk | | Post | | |  |  |
| Energy (kcal) | | | 1732 ± 557 | | 1840 ± 618 | | 1766 ± 521 | | 0.98 | | 1840 ± 557 | | 1720 ± 532 | | 1704 ± 541 | 0.17 | | |
| Carbohydrate (g) | | | 224 ± 72.3 | | 236 ± 93.3 | | 239 ± 78.7 | | 0.17 | | 178 ± 191 | | 216 ± 81.6 | | 189 ± 107 | **0.00*** | | |
| Lipids (g) | | | 58.3 ± 21.3 | | 62.4 ± 21.2 | | 61.4 ± 20.6 | | 0.60 | | 64.3 ± 19 | | 57.1 ± 22 | | 59.3 ± 21.6 | 0.93 | | |
| Protein (g) | | | 77.5 ± 32.2 | | 83.9 ± 37.3 | | 85.4 ± 35.9 | | 0.58 | | 85.3 ± 35.6 | | 85 ± 29.1 | | 80.8 ± 27.4 | 0.66 | | |
| FS (g) | | | 17.7 ± 7.65 | | 18.3 ± 6.60 | | 17.2 ± 8.31 | | 0.41 | | 19.9 ± 6.57 | | 19.3 ± 7.96 | | 16.9 ± 6.01 | 0.89 | | |
| MF (g) | | | 13.8 ± 6.25 | | 15.3 ± 6.08 | | 15.3 ± 6.04 | | 0.49 | | 16 ± 5.49 | | 14.2 ± 6 | | 15.3 ± 6.86 | 1.00 | | |
| PF (g) | | | 8.64 ± 4.25 | | 11.5 ± 7.57 | | 9.90 ± 5.21 | | 0.83 | | 9.62 ± 4.58 | | 8.60 ± 3.93 | | 8.79 ± 3.92 | 0.47 | | |
| COL (mg) | | | 298 ± 219 | | 224 ± 244 | | 322 ± 192 | | 0.62 | | 349 ± 185 | | 370 ± 204 | | 329 ± 192 | 0.99 | | |
| Fiber (g) | | | 13.1 ± 6.60 | | 16.1 ± 7.91 | | 15 ± 7.02 | | 0.89 | | 14.2 ± 10.1 | | 14.8 ± 6.74 | | 15.7 ± 5.62 | 0.63 | | |

Data are presented as mean ± standard deviation according to FTO rs9939609 genotype (TT and TA). SF: saturated fat; MF: monounsaturated fat; PF: polyunsaturated fat; Col: cholesterol. * indicates differences between genotypes across the intervention period (repeated-measures ANOVA, p < 0.05).

**REFERENCES**

Kim DY, Jung SY. Effect of Aerobic Exercise on Risk Factors of Cardiovascular Disease and the Apolipoprotein B / Apolipoprotein A-1 Ratio in Obese Woman. *Journal of Physical Therapy Science*. 2014;26(11):1825-1829. doi:10.1589/jpts.26.1825

López-Bermejo A, Petry CJ, Díaz M, et al. The association between the FTO gene and fat mass in humans develops by the postnatal age of two weeks. *J Clin Endocrinol Metab*. 2008;93(4):1501-1505. doi:10.1210/jc.2007-2343
